# Suitability of European climate for the Asian tiger mosquito *Aedes albopictus*: recent trends and future scenarios

**DOI:** 10.1098/rsif.2012.0138

**Published:** 2012-04-25

**Authors:** Cyril Caminade, Jolyon M. Medlock, Els Ducheyne, K. Marie McIntyre, Steve Leach, Matthew Baylis, Andrew P. Morse

**Affiliations:** 1 School of Environmental Sciences, University of Liverpool, Liverpool, UK; 2 Emergency Response Department, Health Protection Services Division, Health Protection Agency, Porton Down, UK; 3 Avia-GIS, Zoersel, Belgium; 4 Liverpool University Climate and Infectious Diseases of Animals (LUCINDA) Group, Institute of Infection and Global Health, University of Liverpool, Liverpool, UK

**Keywords:** *Aedes albopictus*, vector-borne diseases, climate change, regional climate modelling, Europe

## Abstract

The Asian tiger mosquito (*Aedes albopictus*) is an invasive species that has the potential to transmit infectious diseases such as dengue and chikungunya fever. Using high-resolution observations and regional climate model scenarios for the future, we investigated the suitability of Europe for *A. albopictus* using both recent climate and future climate conditions. The results show that southern France, northern Italy, the northern coast of Spain, the eastern coast of the Adriatic Sea and western Turkey were climatically suitable areas for the establishment of the mosquito during the 1960–1980s. Over the last two decades, climate conditions have become more suitable for the mosquito over central northwestern Europe (Benelux, western Germany) and the Balkans, while they have become less suitable over southern Spain. Similar trends are likely in the future, with an increased risk simulated over northern Europe and slightly decreased risk over southern Europe. These distribution shifts are related to wetter and warmer conditions favouring the overwintering of *A. albopictus* in the north, and drier and warmer summers that might limit its southward expansion.

## 1. Introduction

The Asian tiger mosquito (*Aedes albopictus*; Family Culicidae) is native to tropical and subtropical areas of southeast Asia. It usually breeds in transient water bodies in tree holes, and shows the ability to colonize human-made containers in urban and peri-urban areas [1]. This species lays drought-resistant eggs, which, in an urban setting, are deposited in a number of containers, including discarded used tyres [2]. In recent decades, this species has invaded many countries globally, owing to the transportation of its drought-resistant eggs with shipments of goods (especially used tyres and plants such as lucky bamboo); the speed of such spread has been proportional to an increase in international trade. In 1967, its distribution area was restricted to some parts of Asia, India and a few Pacific islands. Since then, it has spread rapidly to Europe, North and South America, the Caribbean, Africa and the Middle East (see electronic supplementary material, table S1). *Aedes albopictus* is ranked as one of the world's 100 most invasive species, according to the Global Invasive Species Database (http://www.issg.org/database/species/search.asp?st=100ss&fr=1&str=&lang=EN).

As well as being a biting nuisance, *A. albopictus* has been linked to the transmission of arboviral and filarial infectious diseases of humans and animals. Its potential to carry a wide range of human pathogens is consequently of wide concern. *Aedes albopictus* can experimentally transmit numerous viruses, including those that cause West Nile fever, yellow fever, St Louis encephalitis, Japanese encephalitis, dengue fever, Rift Valley fever and chikungunya fever, among others [3–6]. It is also the vector of *Dirofilaria immitis*, a parasitic round worm that causes heartworm in dogs, and less frequently in cats, wolves, foxes and coyotes [7]. It was responsible for the chikungunya outbreak that occurred in 2005–2006 on the French Island of La Réunion. Mutated strains of the chikungunya virus were being transmitted by *A. albopictus* particularly well during this episode [8]. It was also the vector of chikungunya fever during the outbreak that occurred in the summer of 2007 in the Italian province of Ravenna, which infected over 200 people [9]. Recently, in September 2010, two cases of chikungunya fever and two cases of dengue fever transmitted by *A. albopictus* were confirmed in the Var French department [10]. In summer 2010, a case of dengue fever was also diagnosed in a German traveller returning from Croatia [11]. Environmental factors might have exacerbated the establishment of *A. albopictus* into new areas, as its survival range and seasonal activity have been shown to be influenced by a combination of climatic and environmental factors such as temperature, humidity, rainfall and photoperiods [3].

Several studies have been carried out to model and map the distribution of *A. albopictus* based on environmental factors [12–15]. However, they do not consider the impact of the changing climate from 1950 to the present on the spread of the vector. Furthermore, they do not consider the large uncertainties related to the employed mosquito distribution model and the different climate models that are used to simulate the future distribution of the mosquito.

Here, we model and map the distribution of *A. albopictus* over Europe based on climatic features, using different modelling approaches, and including the proviso that the mosquito has already been introduced. A major novelty of this study is the use of three different distribution models to map the climatic suitability of *A. albopictus* for both the current climate and future climate projections using a set of steps within the modelling process. First, differences and similarities across the three different model outputs driven by climate observations are discussed and validated against field-based observations (mosquito absence/presence) for Europe. We also compare how the recent observed climate change context might have favoured mosquito establishment over Europe. In a second step, these distribution models are driven by the simulations of the ensemble of 10 regional climate models (RCMs) to evaluate how suitability for the mosquito might change in the near future (e.g. 2030–2050). This ensemble includes the most up-to-date RCMs that are routinely run by the biggest climate centres in Europe to study climate change (UK Met Office, Météo-France, etc.). This ensemble has been driven by the SRESA1B emission scenario and provides the fine spatial scale information that is required for impact studies. Using an ensemble of RCMs (instead of a single model) takes into account the uncertainties in future climate projections. This framework has been recently successfully applied to model bluetongue transmission risk over Europe [16], but this has not yet been used to map the future distribution of *A. albopictus*, as most of the former published scenarios rely on a single climate model [15] or a single distribution model [14]. Finally, the uncertainties related to the method (vector distribution model) and to the different selected RCMs (future climate model spread) are investigated to make recommendations at the country level.

## 2. Material and methods

### 2.1. Datasets

The observed distribution of *A. albopictus* in Europe is derived from the ECDC/VBORNET dataset which has been collected since 2009 within the ECDC/VBORNET network [17]. The maps are updated quarterly based on confirmed presence and absence information from the broad entomological community. Input of data from experts is possible via the VBORNET website (www.vbornet.eu). Absence and presence data of the mosquito are available at the regional administrative level (NUTS3 or LAU1 dataset; for 52 states or microstates, members of the European Union, located in Europe or close to it). The observations used in this study are based on updated data from December 2011.

A high-resolution (25 km^2^) gridded climate dataset has been developed for Europe based on station measurements [18] within the EC FP6 ENSEMBLES project framework [19]. It provides information on important climate impact variables, including rainfall, temperature, minimum and maximum temperature for the period of 1950–2009 at daily and monthly temporal resolution. This observed climate dataset (EOBS hereafter) was used to estimate the recent climate envelope of *A. albopictus* in Europe.

Regional scenarios for climate change impact assessments require finer spatial resolution than those provided by general circulation models (GCMs) that have a coarse resolution (about 300 km^2^). The ENSEMBLES European project provides improved RCMs, for both recent past (1961–2000) and future climate scenarios (1950–2050). Models covering the European domain with a regular 0.25° step consistent with the observation grid were retained. Two ensembles of simulations have been carried out, the control experiment (SimCTL) and the Scenario experiment (SimA1B). In the SimCTL experiment (1961–2000), all RCMs are forced at their boundaries by the ERA40 reanalysis [20]. Observed external forcing (greenhouses gases, solar, volcanic, aerosols) is applied to all RCMs. In the SimA1B experiment (1961–2050), the RCMs are forced at their boundaries by a GCM with a coarser resolution (about 300 km^2^) forced by the SRESA1B emission scenario (median scenario in terms of CO_2_ emissions [21]). Different GCMs were used to drive the RCMs according to this plan: http://ensemblesrt3.dmi.dk/.

The 10 selected RCMs (and the related operational centre which ran the experiments) are: C4IRCA3 (Met Éireann, Ireland), CNRM-RM4.5 (CNRM, Météo-France), DMI-HIRAM5 (DMI, Denmark), ETHZ-CLM (ETHZ, Switzerland), ICTP-RegCM3 (ICTP, Italy), KNMI-RACMO2 (KNMI, The Netherlands), METO-HC-HadRM3.0 (Met Office, UK), MPI-M-REMO (MPI, Germany), OURANOSMRCC4.2.1 (OURANOS, Canada), SMHIRCA (SMHI, Sweden).

Only the SimA1B future scenario ensemble was considered in this study. Simulated precipitation and temperature outputs for each RCM have been mean bias corrected with respect to the EOBS dataset over the 1990–2009 reference period (see the electronic supplementary material for further details).

### 2.2. Models

#### 2.2.1. Overwintering

Different climatic thresholds were first considered to define the ability of the mosquito to survive European winters based on Medlock *et al*. [12]. Totally, suitable overwintering conditions were defined for mean annual rainfall (AR) above 700 mm and mean January temperatures (*T*_Jan_) above 2°C. Overwintering conditions for a low (defined as 600 mm < AR < 700 mm and 1°C < *T*_Jan_ < 2°C), medium (defined as 500 mm < AR < 600 mm and 0°C < *T*_Jan_ < 1°C) and highly unsuitable scenario (defined as AR < 500 mm and *T*_Jan_ < 0°C) have been investigated and are discussed in the electronic supplementary material. We retained the later scenario as a standard to mask the areas that would be unsuitable for the mosquito for two of the following mapping methods (this is consistent with results shown in [3,15,22–24]). For a more detailed discussion about the overwintering of *A. albopictus*, see the electronic supplementary material.

Three models were used for mapping the distribution of *A. albopictus*:

#### 2.2.2. Model 1. Establishment criteria based on mean annual temperature and overwintering after the Geographic Information System (GIS)-based model developed by Kobayashi *et al.* [25]

The aforementioned overwintering criterion (suitability for *T*_Jan_ > 0°C and AR > 500 mm) was combined with mean annual temperatures to define basic climate suitability zones for the mosquitoes. Totally suitable conditions were defined for mean annual temperature above 12°C. A high, moderate and low risk was then defined for mean annual temperature ranging from 11°C to 12°C, 10°C to 11°C and 9°C to 10°C, respectively. The selection of these thresholds was based on the analysis of Kobayashi *et al*. [25], which showed that *A. albopictus* was relatively well established in Japan for mean annual temperatures above 11°C, while establishment was more stable for annual temperatures above 12°C. Areas in North America where annual temperatures were above 11°C also strongly corresponded to the observed pattern of the distribution of *A. albopictus* in the USA. Further, this is also consistent with the European climatic envelope for *A. albopictus* as shown in Fisher *et al*. [15] and ECDC [14].

#### 2.2.3. Model 2. Multi-criteria decision analysis after ECDC [14]

**Figure 1.**
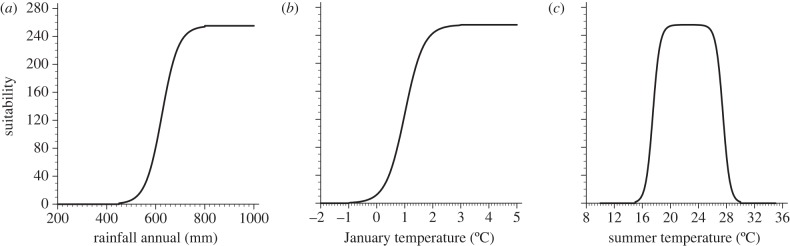
Sigmoidal functions that are employed to relate *A. albopictus* suitability (ranging from 0 to 255) to climate predictor variables such as (*a*) annual precipitation, (*b*) January temperature and (*c*) summer temperature. This is carried out for model 2. For annual precipitation, suitability is dropped to zero when rainfall is lower than 450 mm, and maximum (255) when precipitation is higher than 800 mm; for January temperature, the suitability is zero when temperatures are lower than −1°C, and maximum when temperatures are higher than 3°C; for summer temperature, the suitability is zero when temperatures are lower than 15°C and higher than 30°C, and maximum between 20°C and 25°C.

AR, January and summer (June–July–August) temperatures were first transformed into an interval ranging between 0 and 255 using sigmoidal functions (figure 1). This model does not include the overwintering criterion. Instead, for annual precipitation, suitability was reduced to zero when rainfall was lower than 450 mm, and maximum when precipitation was higher than 800 mm; for summer temperatures, suitability was zero when temperatures were lower than 15°C and higher than 30°C, and maximum between 20°C and 25°C; for January temperatures, suitability was zero when temperatures were lower than −1°C, and maximum when temperatures were higher than 3°C. The three parameters were then linearly combined (arithmetic average) to define the suitability for *A. albopictus*. The suitability was finally arbitrarily rescaled to range between 0 and 100.

#### 2.2.4. Model 3. GIS-based seasonal activity model after Medlock et al. *[*12*]*

This model combines the aforementioned overwintering criterion with weekly temperatures and photoperiods to simulate the weeks of activity of *A. albopictus* between the onset of hatching and the autumn egg diapause. Photoperiod was calculated based on the difference between sunrise and sunset for each grid point of the climate dataset grid (using astronomical equations from the National Oceanic and Atmospheric Administration). First, the aforementioned overwintering criterion was employed to mask the areas where the mosquito would not be able to survive. Then, the start of spring hatching and autumn egg diapause was computed based on the medium scenario: the onset of hatching starts when spring temperature and photoperiod are above 10.5°C and 11.25 h, respectively. The autumn diapause occurs for a temperature threshold of 9.5°C and a photoperiod threshold of 13.5 h.

#### 2.2.5. Model validation

The performances of different models in reproducing the observed distribution of *A. albopictus* were evaluated using the area under the receiver operating characteristic (AUC), a threshold independent quality criterion [26]. The AUC is equal to the probability (ranging from 0 to 1) that a classifier will rank a randomly chosen positive instance (presence location) higher than a randomly chosen negative one (absence location). Useful predictive models have an AUC of about 0.7, excellent models would be above AUC ≥ 0.9 and a random method would have an AUC ≤ 0.5. All model outputs were linearly rescaled between 0 and 1 before being compared with the observed absence/presence data. Spatial correlations between the different distribution models are also investigated.

## 3. Results

### 3.1. Recent trends

**Figure 2.**
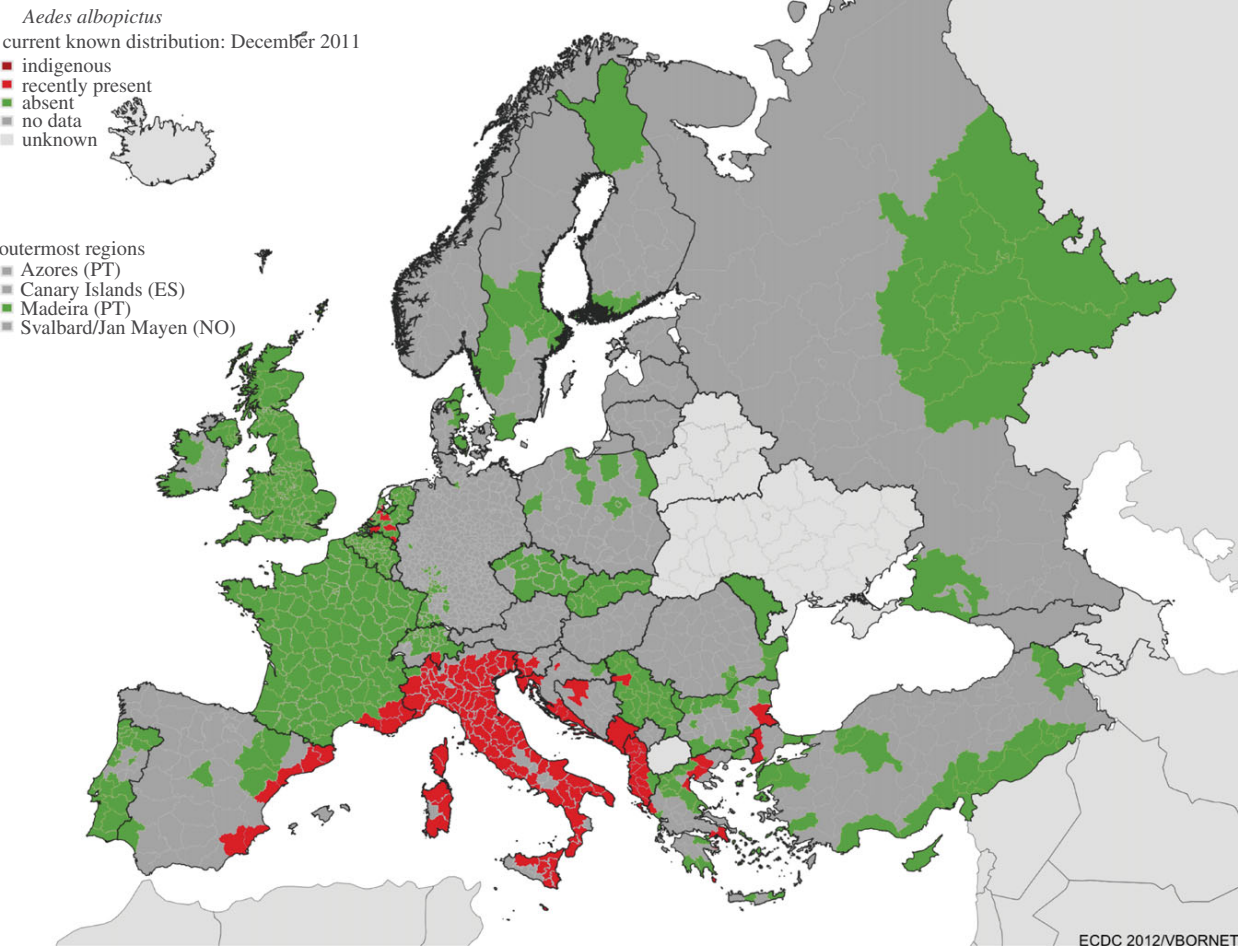
Known distribution of *A. albopictus* based on field observations from the ECDC/VBORNET project (December 2011). Dark red denotes established: the species is observed in at least one municipality of the shown administrative unit for at least 5 years counting back from the ‘distribution status date’. Red, recently present: the species was observed at least in one municipality during the last 5 years. Green, absent: surveys and studies on mosquitoes were conducted during the last 5 years and no specimens were reported. Medium grey, no data: no data over the last 5 years are available to local experts. Light grey, unknown: no information is available on the existence of studies on mosquito fauna over the last 5 years.

The recent observed distribution of *A. albopictus* based on field measurements is shown in figure 2. The species is mainly abundant around the coasts of the Mediterranean and the Adriatic. More precisely, *A. albopictus* has been reported over the eastern coast of Spain, southeastern France, Corsica, Sardinia, Sicily, most of Italy, southern Switzerland, the coast of Slovenia, the coast of Croatia, northwestern Bosnia and Herzegovina, most of Montenegro and Albania, northwestern Serbia, the western coast of Greece, southeastern Bulgaria and in Turkey near the Greek border. The species has also been sporadically observed in several used-tyre storage centres in northern France, Belgium and The Netherlands (here, also in greenhouses) since 1999, as well as on parking areas in southwestern Germany near the French/Swiss border (2007 and 2011). In most of these locations, the mosquito has not established or has been eliminated (elimination is ongoing in The Netherlands). Thus, these findings do not appear on figure 2, where only current established populations are considered. Whether the recent finding in Germany corresponds to an established population is not yet known (see electronic supplementary material, table S1 and references). The UK, Portugal, Czech Republic, Slovakia and Moldova appear to have no *A. albopictus*.

**Figure 3.**
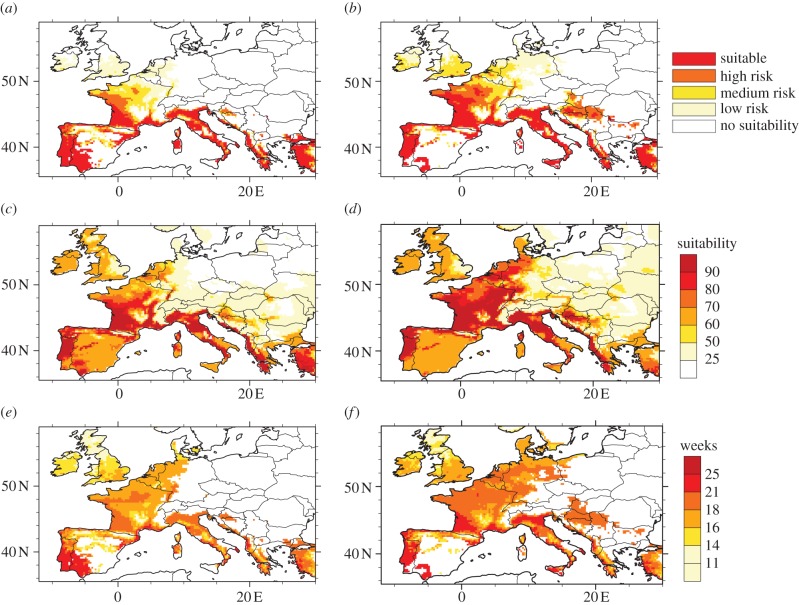
Observed climate suitability of *A. albopictus* based on different models (rows) and for two different time periods (columns). (*a*,*b*) The climate suitability is calculated based on model 1 for (*a*) 1960–1989 and (*b*) 1990–2009. (*c*,*d*) The climate suitability is based on model 2. This is carried out for (*c*) 1960–1989 and (*d*) 1990–2009. (*e*,*f*) Weeks of adult mosquito activity for (*e*) 1960–1989 and (*f*) 1990–2009 based on model 3. See §2 for further details.

Figure 3 depicts the changes in simulated climate suitability for *A. albopictus* based on different models, provided it has been previously introduced. Considering model 1, southwestern and southeastern France, Portugal, the northeastern and northwestern coasts of Spain (including the south west, Corsica and Sardinia), northern Italy and its western coasts, the eastern coasts of the Adriatic Sea and western Turkey appear to be highly suitable for the establishment of *A. albopictus* over the 1960–1989 period (figure 3*a*). During the last two decades (1990–2009), climate suitability increased over France, Italy, the southern UK and it spread over central Europe (Benelux and western border of Germany), western Hungary, the Balkans (Croatia, the northern part of Serbia and Montenegro, Bosnia and Herzegovina) and Sicily, while it decreased over southern Spain and Sardinia (figure 3*b*). These changes are mainly related to observed warmer winter temperatures over northwestern Europe, while the decrease in suitability can be attributed to drier conditions over southern Spain and Sardinia (see electronic supplementary material, figure S4).

Similar results are highlighted over Western Europe using model 2. The hot spots for the establishment of the tiger mosquito in Europe can also be seen over southern France, Sardinia, Corsica and Italy, the coasts of the Adriatic, Spain and Portugal for the period of 1960–1989 (figure 3*c*). However, the simulated suitability pattern is different over the UK and is significantly less over southern Spain and Portugal with respect to model 1. This is related to the inclusion of continuous summer temperatures and AR in model 2 and the fact that no strict overwintering criterion has been included. Indeed, the climate suitability decreases over eastern England as the area is too dry and not warm enough in summer, whereas the differences in southern Spain and Portugal can be related to too dry and warm summer conditions (see electronic supplementary material, figure S4). The risk then spreads and increases over France, northern Italy, central Europe (Benelux and western Germany) and the Balkans during the last two decades (figure 3*d*).

Finally, the simulated periods of activity of *A. albopictus* are compared between the periods 1960–1989 and 1990–2009 using model 3. The areas where the mosquito has the longest activity (about five months) are southern France, the northwestern coast and the southwest of Spain, Portugal, northern Italy, the eastern coast of the Adriatic and western Turkey for the period 1960–1989 (figure 3*e*). The mosquito is simulated to be active for four months over Benelux and western Germany, and between three and four months over the southern UK. The annual activity window of *A. albopictus* has generally lengthened over Europe from 1990 to 2009 (figure 3*f*). The most significant increase can be seen over northern Italy, southeastern and southwestern France, Sicily and the northern coasts of Spain. As the weekly activity of the mosquito is combined with the standard overwintering criterion, the Balkans and central Europe show potential activity of the mosquito at about 18–20 weeks, while this disappears over southern Spain and Sardinia for this period.

All models provide realistic patterns with respect to the observed distribution of *A. albopictus* (figure 2). The models’ predictive performances are good as the AUC scores generally range from 0.63 to 0.7 (table 1 and figure S8 in the electronic supplementary material). models 1 and 3 are significantly spatially correlated (*r* ≈ 0.9, *p* < 0.01; table 2) as they both include an overwintering mask criterion. The spatial correlations between model 2 and models 1 and 3 are also relatively high (*r* ≈ 0.8). Portugal is simulated to be climatically suitable for the establishment of *A. albopictus*, while it has not been observed there yet. The climate suitability regions also cover most of France, the Benelux and western Germany for the period of 1990–2009, while in reality, mosquito presence is more restricted to southeastern France and it has been observed over smaller areas in Belgium and The Netherlands (figure 2). As a reminder, this study concerns only the sustainability of potential climatic conditions for the *A. albopictus* mosquito in Europe. The vector needs to be introduced first before it can settle properly in a given country. For example, Greece appears to be a suitable country for the mosquito to survive in before 1989, but was first detected in 2005.

**Table 1.** Evaluation of the different model performance based on the area under the curve for the receiver operating characteristic (AUC). All method outputs have been rescaled between 0 and 1 and compared with binary absence/presence observations obtained from the ECDC/VBORNET project. The analysis has been carried out only for the regions where the presence or absence of *A. albopictus* has been reported (figure 2).

	1960–1989	1990–2009	2005–2009
model 1	0.69	0.65	0.68
model 2	0.69	0.64	0.67
model 3	0.65	0.61	0.63

**Table 2.** Spatial correlation between the three different models employed for different time periods. All correlations are significant at the 99.9% confidence interval based on the standard Pearson *r*-test.

1960–1989	*r*_1/2_ = 0.77	*r*_1/3_ = 0.92	*r*_2/3_ = 0.78
1990–2009	*r*_1/2_ = 0.75	*r*_1/3_ = 0.92	*r*_2/3_ = 0.75

### 3.2. Future climate scenario

**Figure 4.**
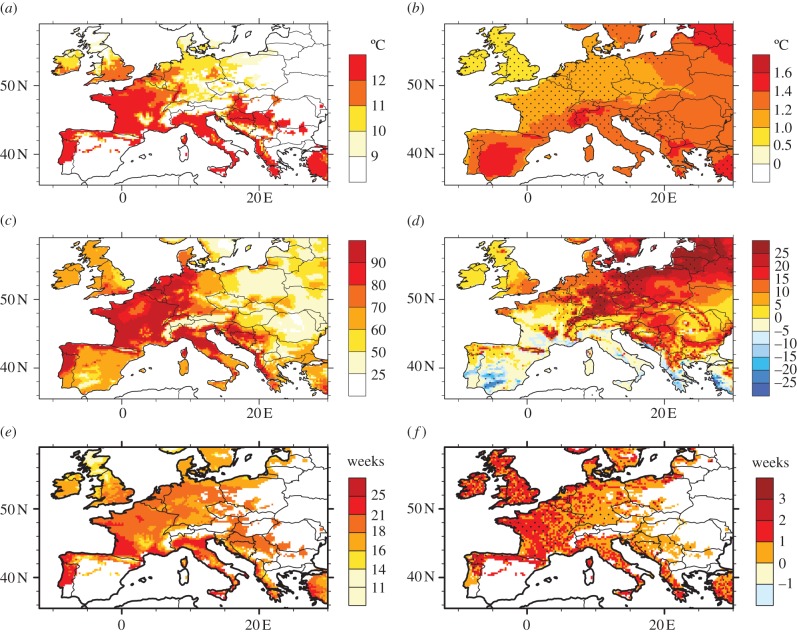
Future climate suitability of *A. albopictus* based on different models (rows). The left column depicts the mean suitability based on the ensemble mean of all RCM-driven projections for 2030–2050. The right column shows the future changes (2030–2050) with respect to the 1990–2009 climatology. The black dots depict the areas where the simulated mean changes are greater than two times the regional climate model ensemble standard deviation. (*a*) Mean future climate suitability based on model 1. (*b*) Annual temperature future change based on model 1. (*c*) Mean future climate suitability based on model 2. (*d*) Future suitability changes based on model 2. (*e*) Weeks of adult mosquito activity for 2030–2050 based on model 3. (*f*) Changes in adult mosquito activity for 2030–2050 based on model 3.

The suitability of future climate for *A. albopictus* is shown in figure 4 based on the ensemble mean of the RCM projections. According to model 1, the future simulated trends are similar to simulated trends for the recent past: increased risk over northwestern/central Europe (Benelux, western Germany, northern part of Switzerland), the southern coast of the UK, and the Balkan countries (Slovenia, Croatia, Bosnia-Herzegovina), and a significant decreased risk over southern Spain, southern Portugal and Sardinia (figure 4*a*). These changes are mainly related to overwintering conditions. Indeed, as the northern European countries get wetter and experience warmer temperatures in winter, this leads to spread in the climate suitability for *A. albopictus*. The simulated decrease in suitability over the southern European countries could be related to simulated drier summer conditions in 2030–2050 (not shown). This feature can be partly seen on figure 4*b*, which shows the mean annual temperature changes for 2030–2050 with respect to 1990–2009. Simulations suggest that temperatures will increase over Europe with a more pronounced warming occurring over southern Europe (especially in summer). Note that the changes in annual temperature simulated by the RCM ensemble mean are robust as they are two times larger than the RCM ensemble spread over most of the European continent (dotted areas on figure 4*b*). Model 2 provides similar results, with a higher risk simulated over the southern UK, the northern coast of Spain, the Balkans and central-western Europe for the future (figure 4*c*). Asymmetric changes in climatic suitability appear clearly on figure 4*d*, contrasting an increase over northern Europe with a slight decrease over the Mediterranean basin. The increase in suitability simulated by the RCM ensemble mean over northern France, the Benelux, Germany and the Balkans and the decrease shown over southern Spain are significant (two times greater than the multi-model ensemble spread, see dotted areas). Changes in the north are linked to increased temperature and precipitation, while the decrease simulated over the Mediterranean basin can be related to drier and warmer conditions in summer (not shown). The impact of future climate change upon the mosquito suitability might then result in its spreading over northwestern central Europe and in its limitation/disappearance around the Mediterranean coasts according to this model. Figure 4*e* highlights the period of activity of the adult mosquito for the future. Simulations suggest that the activity window of the mosquito will generally lengthen over France, Corsica, the northern coasts of Spain, southern UK, Italy and the Adriatic coasts, the Balkans, Benelux and Germany (figure 4*f*). The larger changes are expected over France, the UK and Spain where simulations suggested that adult mosquito activity would lengthen by about two weeks. Mosquito activity could drop to zero over southern Spain and southern Portugal as these areas are generally too dry in future climate scenario simulations.

The increase in suitability over the southern UK, northern France, central and western Europe and the Balkans, and the decrease simulated over southern Spain and Portugal is generally a common feature across all RCM scenarios (electronic supplementary material, figure S5–S7). However, the patterns differ across the various RCM projections. Whereas the MPI-M-REMO and OURANOSMRCC4.2.1 models do not exhibit significant changes (except over the Balkans) with respect to the 1960–1989 period (low scenario), the UK Met Office (METO-HC-HadRM3.0) and the ETHZ-CLM models simulate the largest modifications, with a large increase in the risk over northwestern/central Europe and the Balkans, and more unsuitable conditions over Spain, Sicily and Sardinia related to large drought conditions simulated by these models over these areas.

**Figure 5.**
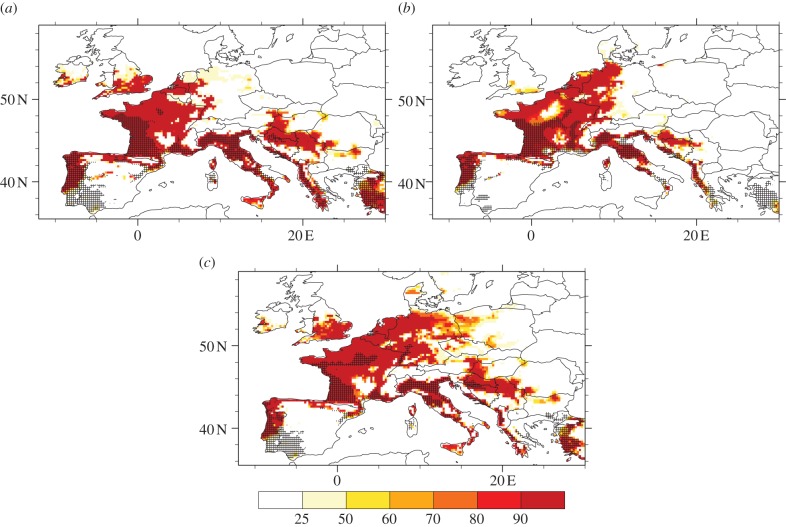
Regions of possible suitability for the *A. albopictus* mosquito according to different time periods and climatic thresholds. The horizontal and vertical striped pattern depicts climatic suitability for the 1960–1989 period based on the EOBS climate observations. The coloured pattern depicts the percentage of RCMs agreeing on the mosquito suitability/presence for the 2030–2050 period. (*a*) Suitability areas are defined for annual rainfall above 500 mm, annual temperatures above 11°C and January temperatures above 0°C (model 1). (*b*) Suitability is defined for model 2 output values above 80%. (*c*) The suitability is defined for adult mosquito activity above 18 weeks (model 3).

The regions of possible suitability for the *A. albopictus* mosquito based on the observed recent climate conditions and future climate projections according to different thresholds are compared in figure 5. This gives insight into multi-distribution model and multi-RCM uncertainties about climatic suitability for *A. albopictus* in the future. Almost all RCM projections simulate an increase in climatic suitability of *A. albopictus* over the southern UK, most of France, Benelux and western Germany and the Balkans (figure 5*a*–*c*). They also corroborate the limitation in its future climatic suitability over southwestern Spain. However, the simulated distribution of the vector slightly differs across the different models and according to the thresholds considered. For example, models 2 and 3 highlight that most of the RCM agree on the spread of *A. albopictus* over a large area covering western Germany (figure 5*b*,*c*), while this seems more restricted to Benelux according to model 1 (figure 5*a*). The simulated future spatial expansion of the mosquito over the Balkans covers a smaller area according to model 2 (figure 5*b*) than models 1 and 3 (figure 5*a–c*). All RCM simulate increased risk covering a larger surface of the southern UK, based on models 1 and 3 (figure 5*a*–*c*), whereas only half of the RCM within the ensemble simulate this feature according to model 2 (figure 5*b*). This also covers a smaller area restricted to the southern coast of the UK.

As a summary, the multi-model approach (using three different distribution models and an ensemble of RCM) shows that the future climatic hot spots for *A. albopictus* are Portugal, the southern UK, the Benelux, western Germany, Slovakia, Cyprus, Macedonia, Bulgaria, Slovakia, Hungary and Turkey.

## 4. Discussion

Distribution maps of *A. albopictus* based on climatic features have been produced for Europe. The three different models highlight that the climatic hot spots for the establishment of *A. albopictus* over Europe are southern France, the northern and northeastern coasts of Spain, Portugal, Italy, the eastern coasts of the Adriatic and western Turkey for the period of 1960–1989. Mosquito climate suitability has significantly increased over the southern UK, northern France, the Benelux, parts of Germany, Italy, Sicily and the Balkan countries (with high values over Slovenia, Croatia and Bosnia-Herzegovina), while it has decreased over southern Spain and Sardinia owing to the drought conditions observed over the 1990–2009 period. Recent climate change might have favoured the presence of *A. albopictus* for most of these countries (the mosquito was detected in France in 1999; Belgium in 2000; Montenegro in 2001; Croatia in 2004, The Netherlands, Slovenia and Bosnia and Herzegovina in 2005; recently found in southwestern Germany in 2007 and in Malta in 2009; see electronic supplementary material, table S1). This is also consistent with the observed spread of this species in Europe as highlighted by Scholte & Schaffner [27].

Future projections highlight similar features to the recent observed trends, with higher risk projected over the southern UK, the Balkans and central Europe (including parts of Germany and Benelux), whereas conditions seem to become more unsuitable over the southern European countries, especially in Spain, Portugal and the Mediterranean islands. These results need to be considered with caution, as there is a significant spread across the different RCM projections. The asymmetry between northern and southern Europe is mainly related to changes in mean climate properties, namely with increased simulated rainfall over northern Europe in winter, and more drought simulated over the Mediterranean basin in summer and a general increase in temperature (more pronounced over northern Europe in winter and southern Europe in summer). These findings based on the ENSEMBLES RCM projections are consistent with the results raised within the IPCC AR4 framework [28]. Indeed, most of the RCM scenarios employed within the IPCC framework highlight an increase in annual mean temperature for 2080–2099 (with respect to 1980–1999) ranging from 2.3°C to 5.3°C in northern Europe and from 2.2°C to 5.1°C in southern Europe. The warming is also more pronounced over northern Europe in winter and southern Europe in summer, and a decrease in the number of frost days is evident in winter over northern Europe. Future rainfall is simulated to increase over northern Europe (especially in winter), whereas more intense drought conditions are likely over the Mediterranean basin in summer. As a consequence, there could be a decreased risk of *A. albopictus* spread as a result of climate change in southern Europe, while this could cause an increase in risk in central-western Europe and the Balkans. However, given that this mosquito is a container species and able to survive in water butts and vases, it would also benefit from water storage in urban areas during drought conditions. The overwintering of eggs can also benefit from an availability of protected areas (including greenhouses), and overwintering of adult female mosquitoes has been observed in Rome since 2006 [29]. Despite slight regional differences, the future hot spots for the establishment of *A. albopictus* broadly agree with published studies [12–15].

This study only concerns the sustainability of potential climatic conditions for the *A. albopictus* mosquito in European countries. However, the vector first needs to be introduced (through used-tyre importation for example) before it could spread or settle properly in different European countries. We have not considered changes in land properties (vegetation, soil types, etc.) that might have a significant impact on mosquito breeding sites. The models employed do not take into account climatic extremes (cold spells, heat waves) that could limit mosquito survival. Future simulations of the effects of climate on mosquito distribution must also be interpreted with great caution, as there are large uncertainties related to the state-of-the-art RCM biases and to the selected emission scenario (SRESA1B). Other important socio-economic factors would ideally need to be considered (changes in international trade, tourism activity). The potential of the vector to carry infectious diseases is obviously affected by the availability of pathogens. Given the recent outbreaks of chikungunya and dengue fever in Europe, effort should be made to conduct surveys for *A. albopictus* in countries that are described as ‘high’-risk for its future establishment (and we also highly encourage a wide surveillance for this invasive species at the European level [30]). These include Cyprus, Bulgaria, Slovakia, Macedonia, Portugal, Turkey, the Benelux, Germany and the UK. There is a potential risk of future establishment in coastal harbour areas for most of these countries (however containers are rarely opened at port and this might reduce the risk).
